# Sarcopenia: Towards a Global Definition and the Potential for Therapeutic Treatments

**DOI:** 10.1093/geroni/igaf122.1280

**Published:** 2025-12-31

**Authors:** Roger Fielding

**Affiliations:** Tufts University, Medford, Massachusetts, United States

## Abstract

The age-related loss of skeletal muscle mass and function, sarcopenia, is associated with well-characterized functional limitations, physical disability, and distal clinically relevant outcomes such as falls, fractures, and death. Underlying these age-related changes are physiological changes in the force/power generating capacity of skeletal muscle that appear to be driven by changes in skeletal contractile protein function, metabolic derangements and alterations in neuromuscular activation. Biologically-relevant age-associated changes in skeletal muscle biology include alterations in gene transcription, mitochondrial stability, anabolic capacity, cellular senescence and metabolic flexibility. Underlying molecular targets have been identified in skeletal muscle that are potential sites for the development of therapeutic interventions. In my presentation, I will provide a state-of-the-art update on key therapeutic targets for components of the sarcopenia syndrome. Two major classes of therapies, selective androgen receptor activation and inhibition of myostatin signaling, have been proposed as targets for treatment of sarcopenia. However, more recently, additional relevant novel pathways have been uncovered that show promise. These include approaches that target skeletal muscle excitation-contraction coupling such as the selective activation of skeletal muscle troponin proteins, and targets that stimulate biogenesis or stability of mitochondria and their upstream activators. I will summarize the available clinical trials data on these emerging novel pathways and discuss the barriers towards regulatory approval for these indications which include the need to develop an international consensus definition of sarcopenia and establishment of treatment guidelines.

